# Integrating genome-wide association and eQTLs studies identifies the genes associated with age at menarche and age at natural menopause

**DOI:** 10.1371/journal.pone.0213953

**Published:** 2019-06-17

**Authors:** Gang Wang, Jian Lv, Xiaoxin Qiu, Yujun An

**Affiliations:** 1 Department of Gynecology, Shandong Provincial Western Hospital, Jinan, Shandong, China; 2 Department of Traditional Chinese Medicine, Shandong Provincial Western Hospital, Jinan, Shandong, China; 3 Department of Obstetrics and Gynecology, Hunan Provincial People’s Hospital Xingsha Branch, Changsha, Hunan, China; 4 Department of Obstetrics and Gynecology, the No.4 Hospital of Jinan, Jinan, Shandong, China; QIMR Berghofer Medical Research Institute, AUSTRALIA

## Abstract

**Objective:**

An early onset of menarche and, later, menopause are well-established risk factors for the development of breast cancer and endometrial cancer. Although the largest GWASs have identified 389 independent signals for age at menarche (AAM) and 44 regions for age at menopause (ANM), GWAS can only identify the associations between variants and traits. The aim of this study was to identify genes whose expression levels were associated with AAM or ANM due to pleiotropy or causality by integrating GWAS data with genome-wide expression quantitative trait loci (eQTLs) data. We also aimed to identify the pleiotropic genes that influenced both phenotypes.

**Method:**

We employed GWAS data of AAM and ANM and genome-wide eQTL data from whole blood. The summary data-based Mendelian randomization method was used to prioritize the associated genes for further study. The colocalization analysis was used to identify the pleiotropic genes associated with both phenotypes.

**Results:**

We identified 31 genes whose expression was associated with AAM and 24 genes whose expression was associated with ANM due to pleiotropy or causality. Two pleiotropic genes were identified to be associated with both phenotypes.

**Conclusion:**

The results point out the most possible genes which were responsible for the association. Our study prioritizes the associated genes for further functional mechanistic study of AAM and ANM and illustrates the benefit of integrating different omics data into the study of complex traits.

## Introduction

Menarche is the first menstrual cycle and signals the possibility of fertility. An early onset of menarche is associated with risks for obesity, type 2 diabetes, cardiovascular disease, breast cancer and all-cause mortality [1]. Menopause is defined as the permanent cessation of menses due to the loss of ovarian follicular activity. Younger age at natural menopause (ANM) is associated with low risk of breast cancer and ovarian cancer, but higher risks of osteoporosis, cardiovascular disease and type 2 diabetes [1]. A Mendelian randomization study has found that later ANM causally increased the risk of breast cancer [2]. These two traits also mark the beginning and the end of a woman’s reproductive life [3].

Genome-wide association studies (GWAS) are capable of identifying the association between target phenotypes and millions of genetic variants. GWAS of age at menarche (AAM) identified 106 loci containing 389 independent signals [4]. GWAS of ANM has successfully identified dozens of significantly associated loci [2, 5, 6]. Most of these loci encode proteins that appear to be involved in DNA repair, immune response and breast cancer processes [2, 5]. However, GWAS can only identify those SNPs strongly associated with the target phenotypes, without pinpointing the target genes and the underlying biological mechanism. For example, the largest GWAS of ANM identified 44 loci containing at least one common variant significantly associated with ANM [2]. However, the significant SNPs in 21 loci were annotated to more than one gene in each locus. It suggested that the specific causal genes remain mostly unidentified.

A large number of genetic variants influences the target phenotypes by causal regulatory effect rather than directly influencing the structure of the protein [7]. Expression quantitative trait locus (eQTL), which is a genetic variant influencing a target gene’s expression, is often used to explain the underlying biological mechanism of significant SNPs identified by GWAS. Previous studies have suggested that in the significant loci, those SNPs which were also eQTLs were more likely to be functional SNPs [8]. Zhu et al. proposed a summary-based Mendelian randomization (SMR) analysis to combine GWAS and eQTL data into a single analysis [7]. SMR integrates GWAS and eQTL data identified from the whole blood tissue to identify potential functionally relevant genes at the significant loci identified in GWAS. Previous studies have shown that whole blood can be a proxy of relevant tissues for various phenotypes and diseases [7, 9].

In this study, we identified genes whose expression levels were associated with AAM or ANM due to pleiotropy or causality, by integrating ANM GWAS data with eQTL data. We conducted a colocalization analysis to identify significant SNPs causally associated with both phenotypes.

## Materials and methods

### AAM GWAS summary dataset

The largest AAM GWAS meta-analysis contained data from ReproGen consortium studies UK Biobank and 23andMe study. Using the 1000 Genomes Project–imputed genotype data in up to ~370,000 European women, 389 independent signals (P < 5 × 10^−8^) were identified for age at menarche [4]. The summary data were downloaded from the following website (http://www.reprogen.org).

### ANM GWAS summary dataset

The largest-scale GWAS meta-analysis summary data of ANM was used in this study [2]. The GWAS meta-analysis conducted with a total sample of 69,360 individuals of European descent from 21 studies identified 44 significant loci. SNPs with the minor allele frequency (MAF) no less than 0.01 and the imputation quality larger than 0.4 were included in the meta-analysis. The summary data were downloaded from the following website (http://www.reprogen.org).

### eQTL dataset

Because the Westra eQTL data [9] had a low coverage of human genes (5,967), in this study we used the Consortium for the Architecture of Gene Expression (CAGE) eQTL data which contained 11,829 unique probes to perform the SMR test [10]. The CAGE study was performed to investigate the genetic architecture of gene expression in peripheral blood in 2,765 European individuals [10]. We set the p-value threshold to be 5×10^−8^ to select the top associated eQTL for the SMR test. After removing those probes where the p-value of the top eQTL was larger than 5×10^−8^, there were 8,144 probes left in the eQTL summary data. The binary summary data can be downloaded from http://cnsgenomics.com/software/smr/#DataResource.

### Genetic correlation

We used stratified linkage disequilibrium score regression (LDSC) to estimate the genetic correlation between AAM and ANM using GWAS summary statistics [11].

### SMR analysis

The method of SMR was fully described in the previous paper [7]. In this study, the phenotypic trait is the outcome (Y), gene expression is the exposure (X), and the top cis-eQTL that is strongly associated with gene expression is used as the instrumental variable (Z). SMR method assumes that the eQTL has an effect on the trait through the gene expression. In brief, there were three models including causality (Z - > X - > Y), pleiotropy (Z - > X and Z - > Y) and linkage (Z_1_ - > X, Z_2_ - > Y, and Z_1_ and Z_2_ are two variants in linkage disequilibrium (LD) in the cis-eQTL region). Since the SMR analysis assumes that the instrument (top cis-eQTL) has a strong effect on the exposure (gene’s expression level), only probes with at least one cis-eQTL at P_eQTL_ (a p-value from the eQTL study indicating the significance of the eQTL associated with the gene expression) smaller than 5×10^−8^ in the cis-eQTL region were included in the eQTL summary data (hg19) [7]. We excluded cis-eQTL with MAF < 0.01 and cis-eQTL in the MHC region because of the complexity of LD patterns in this region [7]. In this study, we tried to identify those genes with causal or pleiotropic effect on AAM or ANM.

To distinguish the causality and pleiotropy models from the linkage model, we conducted the heterogeneity in dependent instruments (HEIDI) test [7]. The HEIDI test considers the pattern of associations using all the SNPs that are significantly associated with gene expression (eQTLs) in the cis-eQTL region (±500kb from the center of the gene probe). The null hypothesis is that there is a single variant affecting trait and gene expression (pleiotropy or causality). The alternative hypothesis is that gene expression and trait are affected by two distinct variants. Under Hardy-Weinberg equilibrium and linkage disequilibrium (LD), b_XY_ (the effect of the gene expression on the trait) estimated at the top associated cis-eQTL (b_XY(top)_) will be equal to that estimated at any of the cis-SNPs in LD that is associated with gene expression. If we define d_i_ = bXY(i)−b_XY(top)_, with b_XY(i)_ being the b_XY_ value of the i-th cis-eQTL, then it is equal to test whether d_i_ = 0. If the number of significant eQTL (excluding the top cis-eQTL) in the cis-eQTL region is m, then we can have a normal vector z_d(i)_ = {z_d(1)_,z_d(2)_,…,z_d(m)_}, where $z_{d(i)}=\frac{d_{i}}{\sqrt{var(d_{i})}}$. To test against d_i_ = 0, we can use the HEIDI test statistic *T*_*HEIDI*_ = z_d_Iz_d_^T^, with z_d_^T^ being the transposed vector of z_d_, and I being an identity matrix, as it is estimated as $T_{HEIDI}=\sum_{i}^{m}z_{d(i)}^{2}$ [7]. SNPs in the cis-eQTL region with a P_eQTL_ > 1.6 × 10^−3^ (equivalent to χ^2^ < 10) were removed to avoid weak instrumental variables according to the original paper [7]. We used P_HEIDI_ > 0.05 to exclude those genes belonging to the linkage model [7]. The SMR software was downloaded from http://cnsgenomics.com/software/smr/#Download.

It was impossible to give a causal conclusion based on only one instrumental variable. In Mendelian randomization studies, multiple uncorrelated instrumental variables (for example, the trans-eQTLs and/or uncorrelated cis-eQTLs) were needed to identify the causality. However, multiple uncorrelated instrumental variables (IVs) were not available in the CAGE eQTL data. In this study, we did not distinguish the causality model from the pleiotropy model.

### Colocalization analysis

Colocalization analysis was used to identify the genetic variants affecting both phenotypes. The method was detailed in the previous paper [12]. In brief, the method based on a hierarchical Bayesian model which can be used to find the region containing a variant that influences both phenotypes. According to the previous paper, there were four models that a given genomic region either 1) contains a genetic variant that influences the first trait, 2) contains a genetic variant that influences the second trait, 3) contains a genetic variant that influences both traits, or 4) contains both a genetic variant that influences the first trait and a separate genetic variant that influences the second trait [12]. It estimated the posterior probability of each model. The threshold of posterior probability equal to 0.9 was used to control the false discovery rate at level 0.1 [12].

## Results

The genetic correlation between AAM and ANM was 0.0079 (p-value = 0.0032). The genome-wide significant level for SMR analysis was P_smr_ < 6.14×10^−6^ (0.05/8,144, Bonferroni test). We identified 98 gene-trait associations with P_smr_ < 6.14×10^−6^. After the application of the HEIDI test, this reduced to 54 gene-trait associations (P_HEIDI_ > 0.05). Those genes which did not pass the HEIDI test may be associated with AAM or ANM due to linkage.

**Fig 1:**
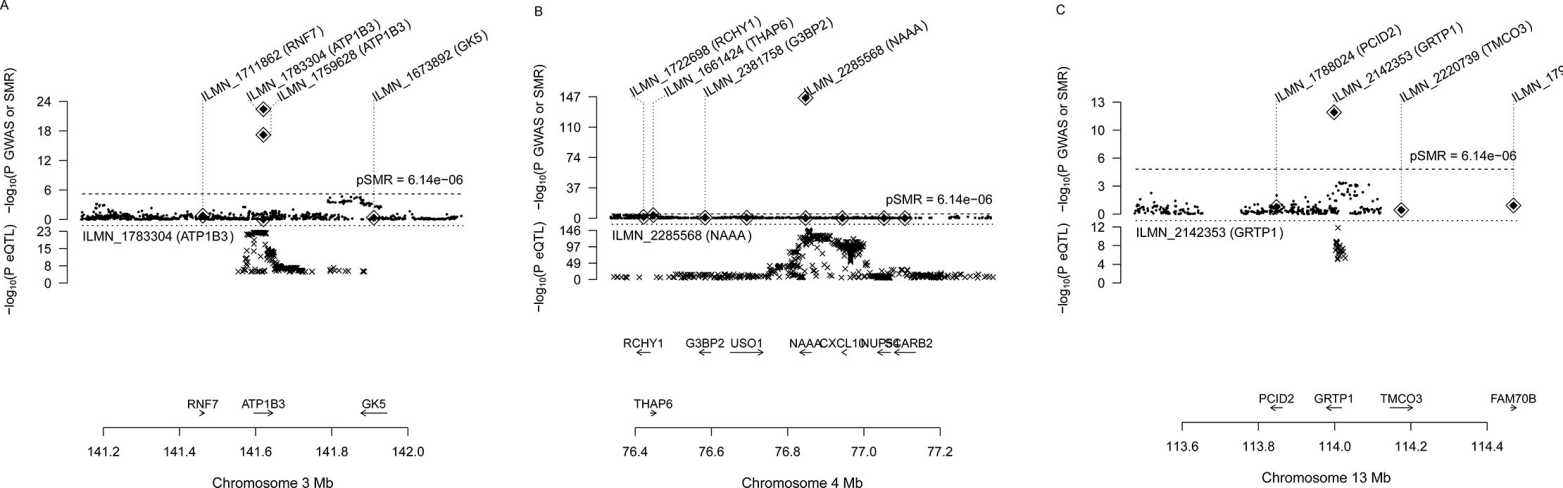
The SMR plots of novel genes associated with AAM. (A) The SMR result at ATP1B3 locus. (B) The SMR result at NAAA locus. (C) The SMR result at GRTP1 locus. In the top plot, black dots represent the p values for the SNPs from the latest GWAS meta-analysis for AAM (Y-axis), diamonds represent the p values for probes from the SMR test. In the bottom plot, the eQTL p values of the SNPs were from the eQTL study (Y-axis).

**Fig 2:**
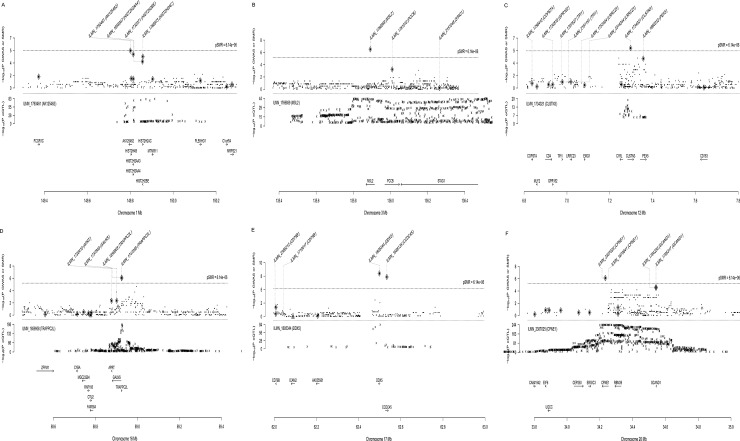
The SMR plots of novel genes associated with ANM. (A) The SMR result at AK125462 locus. (B) The SMR result at MSL2 locus. (C) The SMR result at CLSTN3 locus. (D) The SMR result at TRAPPC2L locus. (E) The SMR result at DDX5 locus. (F) The SMR result at CPNE1 locus. In the top plot, black dots represent the p values for the SNPs from the latest GWAS meta-analysis for AAM (Y-axis), diamonds represent the p values for probes from the SMR test. In the bottom plot, the eQTL p values of the SNPs were from the eQTL study (Y-axis).

We identified 31 genes associated with AAM (Table 1) and 24 genes associated with ANM due to pleiotropy or causality (Table 2). Three (ATP1B3, NAAA, and GRTP1) among of the 31 genes associated with AAM can be considered as novel genes, i.e. no previously identified SNP reported as genome-wide significant in the primary GWAS paper in the cis-eQTL region of the probes (Fig 1). Among the 24 genes associated with ANM, seven genes (MSH6, TLK1, SYCP2L, BRCA1, PGAP3, DIDO1, and DDX17) were previously annotated to be responsible for the association based on distance, biological function, eQTL effect and non-synonymous SNP in high LD. We also identified 6 new genes (AK125462, MSL2, CLSTN3, TRAPPC2L, DDX5, and CPNE1) where there was no significant SNP (p < 5×10^−8^) in the cis-eQTL region of the probes (Fig 2). C17orf46 was the only gene identified to be associated with both phenotypes.

**Table 1:** Genes identified by SMR analysis for AAM.

probeID	Chr	Gene	topSNP	topSNP_bp	p_GWAS	p_eQTL	b_SMR	p_SMR	p_HEIDI	Gene^a^	Gene^c^
ILMN_1869109	1	NUCKS1	rs823094	205689807	4.83E-08	3.39E-38	-0.064	8.24E-07	0.24	NUCKS1	NUCKS1/ RAB7L1/ PM20D1
ILMN_1765061	2	OXER1	rs12617390	42985395	1.25E-07	4.61E-31	0.073	2.45E-06	0.46	OXER1	OXER1
ILMN_1659854	2	PRPF40A	rs7592669	153550668	7.80E-12	2.08E-10	0.16	3.75E-06	0.98	PRPF40A	NA
ILMN_1783304	3	ATP1B3	rs2115935	141616198	0.5	3.82E-23	-0.010	3.82E-23	1.00	NEW^b^	NA
ILMN_1732452	3	MAPKAPK3	rs13096264	50678280	3.26E-10	5.31E-47	-0.070	1.37E-08	1.00	DOCK3	MAPKAPK3
ILMN_1752631	3	CGGBP1	rs9814057	88214472	4.67E-09	1.86E-107	-0.043	2.75E-08	0.98	C3orf38	CGGBP1
ILMN_1744471	3	ZNF654	rs7653652	88189341	3.56E-09	8.55E-79	-0.050	3.17E-08	0.95	C3orf38	CGGBP1
ILMN_2285568	4	NAAA	rs4859572	76857388	0.5	1.90E-146	0.0040	1.90E-146	0.51	NEW^b^	NA
ILMN_1749409	6	HLA-F	rs3870968	29647149	1.16E-09	4.51E-37	0.073	6.40E-08	0.09	HCG4	NA
ILMN_1697309	7	NCF1	rs2267812	74138121	1.87E-13	8.72E-42	-0.088	1.54E-10	0.13	GTF2I	NA
ILMN_2112988	7	NCF1C	rs2267812	74138121	1.87E-13	2.41E-31	-0.10	7.10E-10	0.16	GTF2I	NA
ILMN_2083333	7	PMS2L5	rs3846966	74113141	3.23E-12	3.35E-22	-0.11	2.10E-08	0.09	GTF2I	NA
ILMN_1788384	9	C9orf5	rs12686736	111888739	1.45E-14	1.59E-74	-0.062	2.24E-12	0.13	TMEM245	TMEM245
ILMN_2191929	9	C9orf6	rs874864	111728718	5.66E-13	6.02E-24	0.11	6.09E-09	0.11	TMEM245	TMEM245
ILMN_2163306	9	FAM120A	rs1055710	96214928	1.35E-08	2.83E-15	0.12	5.46E-06	0.86	FAM120A	NA
ILMN_2377829	10	NANOS1	rs671736	120811073	4.38E-08	3.49E-88	-0.043	2.37E-07	0.16	EIF3A	NANOS1
ILMN_1665964	11	GAB2	rs901105	77924607	3.51E-13	8.11E-13	0.16	3.99E-07	0.78	GAB2	GAB2
ILMN_1767642	11	C11orf46	rs7926666	30363101	6.58E-08	8.15E-34	-0.066	1.31E-06	0.24	C11orf46	C11ORF46
ILMN_2094106	11	HSD17B12	rs7118906	43817320	3.67E-07	1.07E-82	0.040	1.62E-06	0.05	MIR129-2	HSD17B12
ILMN_1695585	12	RPS26	rs1131017	56435929	3.25E-07	0	0.019	7.70E-07	0.36	RPS26	NA
ILMN_2142353	13	GRTP1	rs4907616	114008744	0.5	1.52E-12	0.016	1.52E-12	0.87	NEW^b^	NA
ILMN_1727271	14	WARS	rs1570305	100808155	2.63E-09	1.08E-226	0.029	9.21E-09	0.11	WDR25	WARS
ILMN_2080760	15	SNX22	rs12102207	64607472	2.15E-10	9.29E-17	-0.12	5.98E-07	0.49	CSNK1G1	TRIP4
ILMN_1724406	16	INO80E	rs4787491	30015337	6.98E-13	4.48E-18	0.13	4.14E-08	0.06	TBX6	MVP/ KCTD13/ INO80E
ILMN_1717565	16	CLEC18A	rs3748388	69974448	3.75E-14	3.89E-09	0.20	3.71E-06	0.10	NFAT5	WWP2
ILMN_2056687	17	C17orf56	rs1048775	79202329	5.78E-08	4.60E-38	0.066	9.46E-07	0.19	SLC38A10	C17ORF56/ AZI1
ILMN_1707391	17	STXBP4	rs244293	53230722	2.26E-13	4.87E-09	0.20	5.35E-06	0.93	STXBP4	NA
ILMN_1715968	19	MLL4	rs17638853	36234652	1.89E-09	1.41E-12	-0.12	5.89E-06	0.23	KMT2B	COX6B1/ SNX26
ILMN_1776188	20	MAP1LC3A	rs4564863	33179367	1.94E-08	8.12E-110	-0.038	9.50E-08	0.25	GGT7	MAP1LC3A
ILMN_1781225	22	C22orf27	rs5753373	31283719	1.42E-11	1.76E-78	0.057	3.57E-10	0.95	OSBP2	MORC2/ FLJ35801/ OSBP2
ILMN_2103591	22	MORC2	rs7284474	31390187	6.47E-11	7.08E-24	-0.11	5.99E-08	0.10	OSBP2	MORC2/ FLJ35801/ OSBP2

topSNP was the most significant SNP in the cis-region of the probe. topSNP_bp was the position of the most significant SNP. p_GWAS was p-value from GWAS. p_eQTL was p-value from eQTL study. b_smr was effect size from SMR test. p_smr was p-value from SMR test. p_HEIDI was p-value from HEIDI test.

a: These genes were annotated by previous AAM GWAS.

b: These genes were considered as novel genes. No SNP in the cis-eQTL region of the probes was identified to be significantly associated with AAM according to the primary GWAS.

c: Genes were identified by previous SMR analysis in the same locus. NA meant that no gene was identified in this locus.

**Table 2:** Genes identified by SMR analysis for ANM.

probeID	Chr	Gene	topSNP_bp	topSNP	p_GWAS	p_eQTL	b_SMR	p_SMR	p_HEIDI	Gene^a^
ILMN_1810915	1	FAAH	46747301	rs12142240	6.60E-09	5.31E-28	0.38	2.24E-08	5.54E-02	RAD54L
ILMN_1716004	1	NSUN4	46806703	rs10489769	8.20E-08	9.38E-77	0.21	1.14E-08	3.25E-01	RAD54L
ILMN_1793461	1	AK125462	149848885	rs1260246	1.60E-06	3.92E-79	0.21	5.91E-06	9.53E-01	NEW^b^
ILMN_1732810	2	SNX17	27644464	rs1728922	1.20E-14	5.12E-23	0.60	5.06E-10	6.58E-02	BRE / GTF3C2/ EIFB4
ILMN_1670096	2	NRBP1	27584666	rs7586601	2.30E-14	1.59E-10	0.94	5.85E-07	1.82E-01	BRE / GTF3C2/ EIFB4
ILMN_1729051	2	MSH6	48018081	rs1800932	3.20E-11	4.44E-47	0.30	1.34E-07	3.86E-01	MSH6
ILMN_1811029	2	TLK1	171871997	rs13004273	5.90E-17	1.67E-10	1.01	1.89E-07	5.00E-01	TLK1 / GAD1
ILMN_1788053	2	SLC25A12	172704291	rs4668414	2.30E-07	8.11E-21	-0.42	4.40E-07	2.27E-01	TLK1 / GAD1
ILMN_1766859	3	MSL2	136518670	rs13433683	1.10E-05	2.00E-44	0.23	3.08E-07	9.18E-01	NEW^b^
ILMN_1779743	6	SYCP2L	10895260	rs6899676	2.20E-19	3.03E-10	1.08	1.14E-06	6.02E-01	SYCP2L / MAK
ILMN_1798804	6	SRPK1	35809776	rs17705020	1.70E-06	7.09E-34	0.28	3.79E-06	5.41E-02	MSH5 / HLA
ILMN_1767642	11	C11orf46	30363101	rs7926666	1.90E-11	8.15E-34	-0.39	1.99E-08	6.12E-02	FSHB
ILMN_1734021	12	CLSTN3	7284301	rs2167285	1.20E-06	9.69E-20	-0.44	2.53E-06	5.06E-02	NEW^b^
ILMN_1654421	12	MPHOSPH9	123634122	rs884548	6.80E-07	2.39E-18	-0.44	7.57E-07	5.77E-01	KNTC1 / PITPNM
ILMN_1859908	16	TRAPPC2L	88927221	rs3826061	2.80E-06	3.93E-198	-0.11	8.13E-07	7.80E-02	NEW^b^
ILMN_1805636	17	PGAP3	37833035	rs2941506	2.00E-09	2.98E-57	-0.28	1.75E-09	6.23E-01	STARD3/ PGAP3/ CDK12
ILMN_2311089	17	BRCA1	41215825	rs3092994	2.80E-10	1.18E-66	0.28	8.81E-11	1.58E-01	BRCA1
ILMN_1700690	17	VAT1	41215825	rs3092994	2.80E-10	4.47E-15	-0.61	1.77E-07	3.21E-01	BRCA1
ILMN_1805344	17	DDX5	62502435	rs1991401	9.60E-07	2.73E-84	0.22	9.84E-09	8.01E-02	NEW^b^
ILMN_1802053	19	ZNF91	23545004	rs296092	1.50E-06	5.82E-118	0.15	8.78E-08	6.96E-01	ZNF729
ILMN_2307025	20	CPNE1	34221155	rs6060524	1.40E-05	5.28E-244	0.10	7.62E-07	1.00E+00	NEW^b^
ILMN_1812934	20	DIDO1	61558775	rs910831	7.10E-10	2.65E-16	0.55	3.19E-08	4.00E-01	SLCO4A1 / DIDO1
ILMN_2371590	22	DDX17	39021522	rs5757187	1.30E-12	1.88E-28	-0.49	9.01E-11	1.00E-01	DMC1/ DDX17
ILMN_1668535	22	JOSD1	39065172	rs3788545	3.60E-12	3.06E-12	0.78	1.46E-07	7.48E-01	DMC1 / DDX17

topSNP was the most significant SNP in the cis-region of the probe. topSNP_bp was the position of the most significant SNP. p_GWAS was p-value from GWAS. p_eQTL was p-value from the eQTL study. b_smr was effect size from SMR test. p_smr was p-value from SMR test. p_HEIDI was p-value from HEIDI test.

a: These genes were annotated by previous ANM GWAS.

b: These genes were considered as novel genes. No SNP in the cis-eQTL region of the probes was identified to be significantly associated with ANM according to the primary GWAS.

To identify more pleiotropic SNPs and genes associated with both phenotypes, we conducted a colocalization analysis. One region was identified to contain a variant influencing both phenotypes with the posterior probability of 0.92 (Table 3). Thirteen regions were considered to influence the two phenotypes through different variants (Table 3). rs3136249, with the largest posterior probability (0.37), was considered to be the causal SNP influencing both phenotypes.

**Table 3:** Colocalization analysis results of AAM and ANM.

chunk	chr	st	sp	PPA_3	PPA_4
162	chr2	47318990	48212562	0.92	0.064
1419	chr15	88370262	90473690	2.5E-17	1.00
1579	chr19	614967	2098015	1.1E-08	1.00
360	chr3	135458294	137370076	4.1E-06	1.00
653	chr6	28918936	29737846	3.3E-05	1.00
799	chr7	92500845	93966036	5.4E-05	1.00
1637	chr20	32819871	34960201	6.7E-05	0.99
656	chr6	31572333	32682429	3.5E-03	0.97
655	chr6	30798697	31568469	6.1E-04	0.94
1682	chr22	19913726	22355640	4.0E-04	0.94
128	chr1	241582668	242070731	2.5E-04	0.93
1644	chr20	42680811	44838112	1.9E-03	0.93
1693	chr22	37570784	39306630	3.0E-04	0.92
1213	chr12	55665948	57543572	3.7E-04	0.91

Chunk was the internal numerical identifier for the segment. chr: chromosome. st: star position. sp: end position. PPA_3 was the posterior probability of model 3. PPA_4 was the posterior probability of model 4.

## Discussion

In this study, we identified 31 genes whose expressions were associated with AAM and 24 genes whose expressions were associated with ANM due to pleiotropy or causality. In total, we identified 9 new genes where there was no significant SNP in the cis-eQTL region of the gene probe. Many of these genes participated in DNA repair, immune response, and breast cancer process [2, 5]. C17orf46 was identified to be associated with both phenotypes by integrating GWAS and eQTLs data. We also found one region with a pleiotropic effect influencing two phenotypes through the colocalization analysis.

Although the previous study performed the SMR analysis with Westra eQTL data which had a low coverage of human genes (5,967) compared to CAGE eQTL data (8,144). Thus the previous study may omit many potential genes. Eleven genes of the 31 genes associated with AAM were successfully identified by using CAGE eQTL data (Table 1).

SMR demonstrated that it was useful to prioritize genes associated with AAM or ANM. SMR tests reduced the multiple hypothesis burdens by testing tens of thousands of genes instead of millions of SNPs [13]. It suggested that SMR was useful in identifying novel genes associated with AAM or ANM.

In this study, we identified 3 novel genes associated with AAM and 6 novel genes associated with ANM. One novel gene DDX5, which is also known as p68, was identified to be associated with ANM. DDX5 is a prototypic member of the DEAD box family of RNA helicases that encompasses multiple functions. DDX5 was highly expressed in a high proportion of breast cancers. Patients with a detectable level of both DDX5 and polo-like kinase-1 (pLK1) often had a poor prognosis [14].

In the significant loci, we redefined the functional genes which were more likely to play important roles in the process of menarche or natural menopause. We presented a list of genes to be followed up in future functional validation experiments. For example, HSD17B12 coding a 17beta-hydroxysteroid dehydrogenase transforms estrone (E1) into estradiol (E2) [15]. E2 is involved in the regulation of the estrous and menstrual female reproductive cycles. However, the previous study annotated the significant SNP to MIR129-2 (Table 1). Fatty acid amide hydrolase (FAAH), the enzyme that breaks down the endocannabinoid anandamide and controls its levels, is regulated by estrogen [16]. The previous study annotated the significant SNP in this locus to RAD54L (Table 2), however, we found that FAAH was more likely to be the causal gene in this locus. Another example is SRPK1, encoding the splicing factor kinase SRSF protein kinase 1, which was highly expressed in basal breast cancer cells [17]. The knockdown of SRPK1 significantly suppressed metastasis of breast cancer cells [18].

Despite the common belief that multiple genes are responsible for controlling the timing of menarche and natural menopause, very few genes have been identified that contain common genetic variants associated with AAM and ANM. In this study, we identified two genes (MSH6 and C11orf46) associated with both traits. rs3136249 is located in the intronic region of MSH6. MSH6, which is a mismatch repair gene, was found to be associated with ANM by the previous study [19]. The colorization analysis showed C11orf46 locus may be associated with both traits with the posterior probability of 0.79. This may be caused by the relatively large posterior probability of model 4 (0.21). However, the function of C11orf46 is unknown, further studies are needed to prove this result.

The present study may have some limitations that should be considered. Although we redefined the functional genes in the significant loci, these genes may be associated with age at natural menopause due to pleiotropy which meant that some of these genes may be not the causal genes. Due to the limitation of the method, we did not distinguish those pleiotropic genes from causal genes. So, further works are warranted to confirm the functional genes and explore the underlying mechanism.

In conclusion, we highlighted the putative functional genes in the significant loci for AAM and ANM. Our study prioritizes the associated genes for further functional mechanistic study of AAM and ANM and illustrates the benefit of integrating different omics data into the study of complex traits. Our study may help to understand the ovarian function and benefit for women’s reproductive health.
